# Real-Time Epidemic Monitoring and Forecasting of H1N1-2009 Using Influenza-Like Illness from General Practice and Family Doctor Clinics in Singapore

**DOI:** 10.1371/journal.pone.0010036

**Published:** 2010-04-14

**Authors:** Jimmy Boon Som Ong, Mark I-Cheng Chen, Alex R. Cook, Huey Chyi Lee, Vernon J. Lee, Raymond Tzer Pin Lin, Paul Ananth Tambyah, Lee Gan Goh

**Affiliations:** 1 Department of Clinical Epidemiology, Tan Tock Seng Hospital, Singapore, Singapore; 2 Duke-NUS Graduate Medical School, Singapore, Singapore; 3 Department of Epidemiology and Public Health, National University of Singapore, Singapore, Singapore; 4 Department of Statistics and Applied Probability, National University of Singapore, Singapore, Singapore; 5 Biodefence Centre, Ministry of Defence, Singapore, Singapore; 6 National Centre for Epidemiology & Population Health, Australian National University, Canberra, Australia; 7 National Public Health Laboratory, Ministry of Health, Singapore, Singapore; 8 Department of Medicine, National University Hospital, Singapore, Singapore; 9 College of Family Physicians, Singapore, Singapore; Fred Hutchinson Cancer Research Center, United States of America

## Abstract

**Background:**

Reporting of influenza-like illness (ILI) from general practice/family doctor (GPFD) clinics is an accurate indicator of real-time epidemic activity and requires little effort to set up, making it suitable for developing countries currently experiencing the influenza A (H1N1 -2009) pandemic or preparing for subsequent epidemic waves.

**Methodology/Principal Findings:**

We established a network of GPFDs in Singapore. Participating GPFDs submitted returns via facsimile or e-mail on their work days using a simple, standard data collection format, capturing: gender; year of birth; “ethnicity”; residential status; body temperature (°C); and treatment (antiviral or not); for all cases with a clinical diagnosis of an acute respiratory illness (ARI). The operational definition of ILI in this study was an ARI with fever of 37.8°C or more. The data were processed daily by the study co-ordinator and fed into a stochastic model of disease dynamics, which was refitted daily using particle filtering, with data and forecasts uploaded to a website which could be publicly accessed. Twenty-three GPFD clinics agreed to participate. Data collection started on 2009-06-26 and lasted for the duration of the epidemic. The epidemic appeared to have peaked around 2009-08-03 and the ILI rates had returned to baseline levels by the time of writing.

**Conclusions/Significance:**

This real-time surveillance system is able to show the progress of an epidemic and indicates when the peak is reached. The resulting information can be used to form forecasts, including how soon the epidemic wave will end and when a second wave will appear if at all.

## Introduction

On 2009-04-24, the World Health Organization (WHO) reported the spread of a novel influenza A (H1N1) strain in the United States and Mexico. Sentinel surveillance which was mainly hospital based had indicated increased numbers of influenza-like-illness (ILI) in Mexico occurring since 2009-03-18 [1]. Over the next months, the virus spread rapidly across the globe, resulting in the WHO declaring a pandemic and advising countries to activate their pandemic preparedness plans [2]. Singapore identified her first imported case of influenza A (H1N1-2009) on 2009-05-27 [3], and the first unlinked cases on 2009-06-19 [4], which indicated community transmission had begun in Singapore.

**Figure 1:**
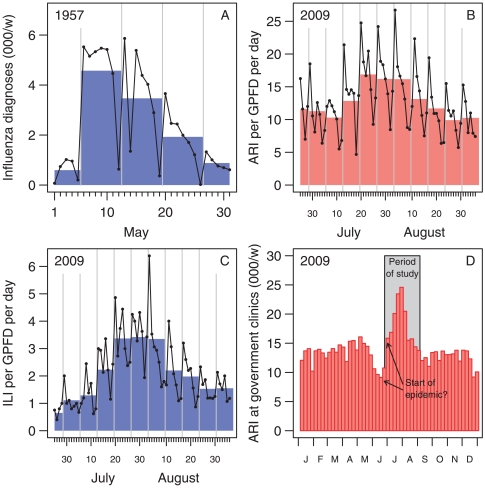
Influenza diagnoses in Singapore in 1957 and 2009 using alternative methods. (A) ILI in government and city council clinics, 1957 [7]. (B) ARI in this GPFD sentinel network, 2009. (C) ILI in this GPFD network, 2009. (D) Weekly ARI in government polyclinics, 2009 [31]. (A–C) Both daily counts (lines) and weekly averages (shaded polygons) are presented. (D) A marked drop in baseline ARI consultations can be seen immediately before the epidemic, complicating the determination of when the epidemic started using this measure.

Singapore experienced all three influenza pandemics of the last century—in 1918, 1957 and 1968 [5], [6]. During the 1957 pandemic, reporting of influenza cases by clinicians provided a reasonably clear indication of daily epidemic activity (Figure 1A) [7]. Influenza-like illness (ILI) has also been used widely as an indicator of influenza activity during non-pandemic epidemics, with ILI reporting by sentinel general practice/family doctor (GPFD) clinics forming the backbone of surveillance systems for influenza in many countries [8]–[14], and these have been used to monitor the current pandemic [15]–[18]. In Singapore, though, acute respiratory illness (ARI) data captured from electronic medical records, as a more general indicator of infectious disease outbreaks, have traditionally been used by health authorities, including during the early part of the current pandemic. However, ILI monitoring can provide an estimate of case numbers and hence attack rates, hospitalisation and case fatality ratios [19], and is more specific for influenza than ARIs.

Data from ILI monitoring can also be used for modelling of influenza epidemics and pandemics [20]–[23]. Modelling can be performed retrospectively to determine the relative importance of community compared to household transmission, or to determine the effect of pharmaceutical and non-pharmaceutical interventions [21], [23]–[26]. Modelling can also be performed in real-time during an epidemic, as proposed by Hall and colleagues, who used mortality data from England and Wales to demonstrate how models could have forecast when epidemic activity would peak during several historical pandemic events [27]. Since H1N1-2009 has low hospitalisation and mortality rates (less than 1% of infected individuals) [28], reporting of ILI from GPFD clinics would potentially provide a more accurate indicator of real-time epidemic activity and progress than hospitalisations and confirmed fatalities.

While data on ARIs are routinely collated and laboratory surveillance of influenza has been in place in Singapore for more than 30 years [29], there is currently no system for monitoring GPFD consults for ILI in Singapore. In order to monitor the epidemic and adjust response plans in real-time, we rapidly developed a system for ILI surveillance, with resulting data and forecasts made publicly available via a website. The purpose of this paper is twofold:

- to describe how the system was developed and used to monitor the progress of the epidemic; and
- to describe how the resulting information was used to perform near real-time forecasting of the course of the epidemic in Singapore.

## Results

**Figure 2:**
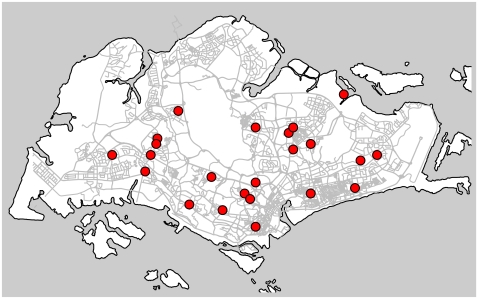
Spot map showing the locations of participating GPFD clinics in Singapore. Most populated parts of the island were represented, the exception being the Woodlands, Sembawang and Yishun areas to the North.

We started the project in early June 2009, shortly after Singapore identified her first imported case of influenza A (H1N1-2009) on 2009-05-27. We sent out mass appeals to 535 e-mail addresses of GPFDs or clinics, and 23 clinics agreed to participate; the locations of the participating GPFD clinics are shown in Figure 2. Four clinics were city or office area practices and the remainder were situated in residential areas across the island.

Figure 1(B,C) shows trends in consultations for ARIs and ILIs from the network. Data submissions started on 2009-06-25, by which time there had been 315 confirmed H1N1 cases (including 87 locally transmitted cases) in Singapore [30]. There was a clear but initially unanticipated weekly periodicity to the data, with lower consultation rates on the weekend and a post-weekend surge in attendances. For descriptive purposes (but not analytical ones), we therefore used weekly averages to provide a smoothed picture of the epidemic trajectory. A comparison between Figure 1B or D and C clearly displays ILI as a better indicator of epidemic activity than ARI. The weekly average ARI consults per doctor in the early epidemic period was between 10 and 15 (Figure 1B), and peaked at 17 in the week ending 2009-07-25, but from this alone it was difficult to determine how much H1N1-2009 epidemic activity there was around the time community transmission was starting; this is compounded by the high baseline rate making the height of the peak relatively low, at just around one and a half times the baseline level. Figure 1D shows the weekly epidemiological data for acute respiratory tract infections in Singapore based on government clinic attendances for ARI [31]. The government clinic ARI data peaked in the week ending 2009-08-01, but, as with our ARI surveillance data, the high levels of background noise make it difficult to ascertain how much community-level infection there was near the start of the epidemic, especially since the epidemic was preceded by a considerable dip in ARI numbers. On the other hand, there was a marked, nearly five-fold increase in our ILI case data, from an average of about 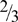 of an ILI per GPFD per day in the week ending 2009-06-27 to a peak of 3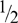 in the week ending 2009-08-01. The highest recorded ILI rate occurred on 2009-08-03 (a Monday) with 6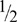 ILIs per family doctor being reported. The sentinel network indicated that the epidemic had peaked around the start of August, and that ILI rates had returned to near baseline levels early in September.

**Figure 3:**
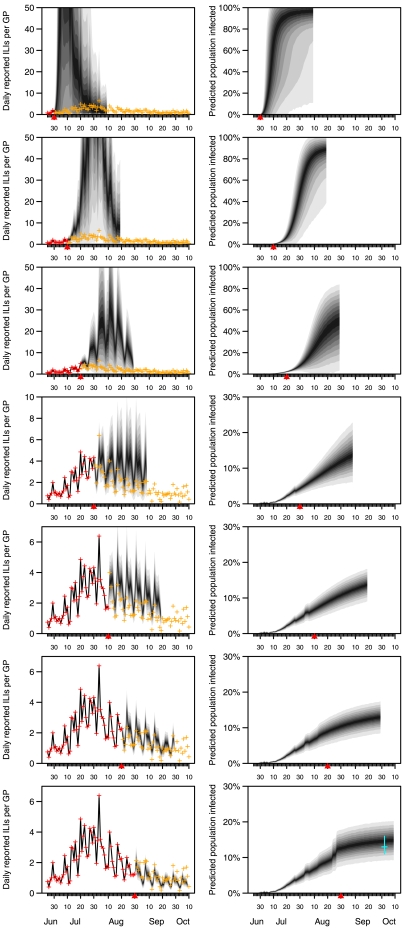
Evaluation of forecasts. (Left) Actual (red and orange crosses) and predicted (grey shaded area) average number of patients presenting with influenza-like illness per day at the average participating GPFD. The information used to form the forecast is indicated by the red crosses. The last day of information used in forming the forecast is indicated with a red triangle. Predictions here (and in the right-hand column) take the form of decreasing credible intervals, with the region spanned by the outermost polygons corresponding to 95% credibility. Orange crosses indicate future data not used in forming the forecasts. (Right) Predicted total number of people who (i) are currently symptomatic, or (ii) have recovered, assuming no pre-existing immunity. The last day of information used in forming the forecasts is indicated with a red triangle. The cyan cross on the bottom panel indicates the age-adjusted estimate of adult seroconversion in the community from an independent study (maximum likelihood estimate and 95% confidence interval, Mark I-Cheng Chen, personal correspondence).

**Figure 4:**
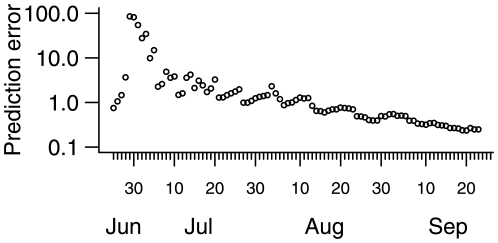
Quantification of predictive error. Posterior absolute deviation between predicted average ILIs per GPFD and observed average, with error averaged over the one week period following the time the forecast is made.

**Figure 5:**
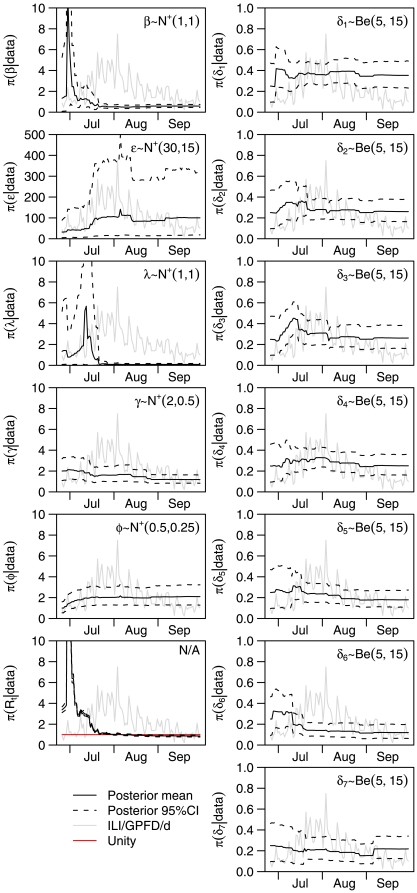
Subjective posterior distributions of parameters and 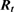 Posterior mean and marginal point-wise 95% credible intervals. The reader's posterior distributions may differ from ours (see refs [32]–[34]). In the background for reference is the number of ILIs per GPFD per day (not to scale). The line of unity is marked on the panel for the effective reproduction number, 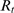; the posterior crosses the line of unity around the day of the peak. Prior distributions for the parameters (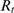 is not a parameter) are indicated on the appropriate panels, using the notation 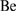 for the beta distribution and 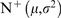 for the modified normal distribution such that if 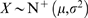 then 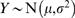 and 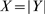. The prior distributions taken for the states were 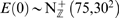, 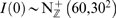 and 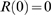 (a Dirac delta prior), where 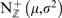 is similar to 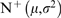 except that its support is the integers, and its mass function at 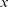 is obtained by integrating the density for 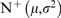 from 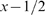 to 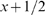.

Predictions of the number of ILIs being seen by our GPFDs and of the total number of people infected are presented in figure 3; animations of these forecasts and of the forecast total number seeking medical attention can be found in the supporting information (ILI/ GPFD /d in video S1, total ILI/d in video S2 and cumulative infections in video S3). These incorporate both population stochasticity and parametric uncertainty. The eventual forecast was that 13% of the population had been infected, with a 95% credible interval of (9%,19%). Initial forecasts were adversely affected by uncertainty in the parameters, caused by the vagueness of the subjective prior distributions we used and the scarcity of information from the data. By the middle of July, the algorithm was correctly forecasting the peak would occur at the start of August, although the magnitude of the epidemic was grossly overpredicted, and the accuracy of the forecast of the time of the peak may have been merely fortuitous. By the end of July, forecasts were stabilising around what transpired to be the eventual data, and by the middle of August, after the peak had come, the forecasts closely foreshadowed the tail of the epidemic. A measure of predictive accuracy is presented in figure 4. By the end of July, predictive error was averaging around 1 ILI per GPFD per day over a one-week time horizon. The sequence of subjective posterior distributions for the parameters and for the effective reproduction number 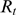 over time are presented in figure 5, although we stress that these are our subjective distributions and do not expect the reader to share them [32]–[34].

## Discussion

We have shown that it is possible rapidly, and at short notice, to deploy a real-time influenza epidemic surveillance system using GPFDs in the absence of an existing system. This is likely to be a workable model in much of the developing world where a significant proportion of primary care is delivered by private practice GPFDs. Firstly, we provide proof of concept that it is feasible, within a month, and with no budget, to establish a protocol for daily data submission for ILI and begin submission. Secondly, we show that processing the data in near real-time—with cases seen each day entered by the following day—can provide graphical trends that describe the progress of an influenza epidemic. Finally, we demonstrate how such data can be used in real-time, and in combination with a process-based model refitted daily, to generate forecasts that can subsequently be verified against actual data as an epidemic unfolds, as is common in other dynamic applications such as weather and finance.

While ILI surveillance is used widely in temperate countries [8]–[13], there are few publications on the effectiveness of ILI surveillance in tropical countries to chart the spread of epidemic influenza, given the high baseline incidence of other non-influenza diseases and minimal seasonal forcing. Evidence is now emerging on the value of such surveillance systems in the tropics [35], and our study shows that ILI surveillance can track epidemic influenza activity in such settings. The slow uptake of influenza surveillance systems for tropical countries may be related to the lack of appreciation for the epidemiology and impact of tropical influenza [36]. Previous work has shown that both non-pandemic (often called “seasonal” in temperate countries in which influenza is associated with winter) and pandemic influenza caused substantial excess mortality in tropical Singapore [5], [37].

In Singapore, influenza activity has traditionally been monitored through a combination of laboratory and ARI morbidity [29]. ARI data reflect the total burden of acute respiratory illness from all causes, often including non-infectious causes such as exacerbations of chronic lung disease which may be environmental in origin. However, it is clear from this study that while both ARI and ILI counts give an indication of when epidemic activity peaks, ILI data provide better resolution of influenza epidemic activity, with the relative magnitude of increase over the baseline being far greater than for ARI data, since influenza activity in the early epidemic phase is masked by the high and obstreperous baseline rates of other respiratory illnesses diagnosed as ARI. The other system for tracking influenza activity in Singapore is based on laboratory confirmed diagnoses of influenza. This is similar to what is done in many countries throughout the world as part of the World Health Organization's Global Influenza Surveillance Network. Monitoring of laboratory confirmed diagnoses picked up an increase in H1N1-2009 isolates among a sub-sample of ILI cases presenting at government polyclinics about one week before the epidemic was apparent in our ILI data (data not shown). However, the advantages of ILI surveillance is that it is much cheaper than laboratory-based surveillance and there are no capacity issues that may limit the number of samples that can be processed daily. In addition, laboratory testing of random samples is less sensitive to changes in absolute numbers of community cases at the peak of the pandemic when the influenza proportion among ILI cases remains relatively steady [31]. ILI surveillance is therefore a cheaper and possibly more effective alternative to traditional laboratory surveillance, especially for resource-poor areas, to obtain reasonable sample sizes.

Setting up such a surveillance network has the secondary benefit of allowing real-time forecasting, which allows more informed policy making. By forecasting the epidemic ahead of time, we allow our forecasts of epidemic activity to be verified against data. We observed during the epidemic that modelling results correctly forecast the timing of peak epidemic activity on some days, but was off by up to a week at other times, though the actual magnitude of the peak was markedly different from early forecasts. We note though that even the relative accuracy of the forecast of the timing of the peak may have been merely fortuitous, and stress that we provide no theoretical results to guarantee this accuracy is repeatable. One particular difficulty we faced was ensuring the predictive accuracy of the system, given the lack of training data and the need to inform policy making as the epidemic unfolded. The results presented herein are therefore almost entirely the same as those presented on-line, including any shortcomings; the only alterations to the model and approach were to allow reporting rates to vary across the week (a change partially implemented part-way through the study) and to remove an adhoc method intended to make the approach more robust to potential changes to the parameters in time (which transpired not to improve matters enough to warrant introducing statistical non-coherencies).

The eventual forecast for the final size of the outbreak was around 13% with a 95% credible interval of (9%,19%). If true, then combined with the rolling out of vaccine and the potential for some additional existing immunity [38] (a possibility we conservatively excluded from the analysis), this figure suggests Singapore is unlikely to experience a large second wave without substantial mutation of the pathogen. The estimate of around 13% corresponds closely to a paired serological study of Singaporean adults which estimated 13% (11,16)%, adjusting for the age distribution of the country, had experienced a four-fold rise in antibody titres (Mark I-Cheng Chen, personal communication). The close correspondence adds considerable confidence to the conclusions of the study.

Further evaluation is underway of the value of retaining a sentinel network permanently in a tropical city-state with year-round non-pandemic influenza transmission and additional bi-annual epidemics. By establishing an avenue for public display of infectious disease forecasts, we hope to build public and institutional confidence in and acceptance of modelling in the context of infectious diseases. To this end, the network was publicised in the local media and the website was made freely available to the general public. This helped provide an additional layer of transparency to reporting of the numbers of people infected with influenza and the relative impact on the wider community. We believe that this contributed to the overall national risk communication strategy and helped to reduce the level of panic and disruption to normal activity feared at the onset of the pandemic.

Several limitations of our work need to be highlighted. Firstly, this system of data collection was fully dependent on the goodwill of participating GPFDs, who received no monetary compensation. We found that we could continue to motivate the participating GPFDs by providing frequent updates based on their aggregated contributions. Although we sent out mass appeals to over 500 e-mail addresses, only 23 GPFDs agreed to participate. The poor response rate could be due to a combination of factors, including:

1. duplicate or invalid e-mail addresses, the former of which could have been addressed by pre-grepping the list, the latter by better book-keeping;
2. spam filtering, which can only really be addressed by using alternatives to e-mail, such as facsimile;
3. lack of publicity on the objectives and importance of our project, which might have been improved by more careful rhetoric in the invitation letter we sent;
4. and reluctance by GPFDs to commit to the burden of data collection during an impending epidemic which was already anticipated to increase workload.

The final premise may be the most critical, and we suggest that some form of financial reimbursement be considered to compensate GPFDs for the effort and time needed to drive data submission in future, as this would likely improve recruitment rates and make such a system sustainable in the long term. Overall, the poor response rate highlights the challenge of recruiting appropriate clinics for any such system, particularly when using e-mails to disseminate such information, and at short notice. However, for a medium-sized city of 4.8 million residents, the network of around 20 GPFDs sufficed to provide considerable information on epidemic progress. Notwithstanding this small number of participating GPFDs, the surveillance system achieved its intended objective of tracking and forecasting influenza epidemic activity in near real-time. The small number of participating GPFDs (estimated to be about 2% of all GPFD clinics in Singapore) may make it difficult to assess if our ILI data are representative of all influenza diagnoses during the epidemic, but this is a limitation common to sentinel GPFD networks for influenza. The potential impact of non-representativeness caused by non-response would not, however, impact the validity of the forecasts, since the methods used for that do not assume the sentinels were selected at random. Other countries have used GPFD networks for surveillance of other viral illnesses [8]–[13], [20], [21], [28] and perhaps the combined lessons from these strategies could be applied more widely internationally.

In hindsight, several aspects of the approach could have been bettered. We did not anticipate the strong day of the week effect on ILI consulting rates, and this had a deleterious effect on predictions, especially when moving to Mondays from Sundays. In mid-July we changed the model to allow different rates at the weekend from the rest of the week, but by mid-August it became clear that the model would fit much better were every day of the week allowed its own reporting rate; this is the model presented herein. Again, in hindsight, it is obvious that there was bound to be sufficient information in the data to be able to estimate the differential reporting rates over the days of the week. Alternative models, such as the Richards model [39], [40], might have proven as or more effective, and certainly could be more parsimonious, than the compartmental model we used, but our experience was that the challenges of developing the software before any data had been collected effectively ruled out deciding on an optimal model to use. As is common in the field of infectious disease modelling, the model we used made many simplifying assumptions (see methods), all of which may potentially have reduced the quality of the forecasts. For instance, the presence of heterogeneous mixing or susceptibility in reality but not in the model may lead eventually to changes to the parameter estimates over time as the routine endeavours to fit a model excluding these effects, but in forming forecasts at an early stage, the future path of parameter estimates is unknown and so forecasts cannot take this into account. In this paper, we have used the term “forecast” *sensu* Keyfitz [41], to indicate the belief we invested in these predictions and the way they were used in contingency planning in some of the authors' institutions. This contrasts with his definition of a projection, which is the extrapolation of past trends without claiming to expect them to match the future. A consequence of this reticence, according to Keyfitz, is that projections cannot be wrong (never being claimed right), while predictions or forecasts are “practically certain” [41] to be in error, and are prone to black swan-type events [42]—accepting this, and excepting the initial predictions, the forecasts we made fared very well (figures 3 and 4). Had we concentrated instead on projecting the epidemic, via a suite of competing models, we might have learned more about the assumptions underlying those models, which would have informed future modelling efforts. A comparison of different projecting approaches, as has been done for seasonal influenza monitoring [43], would therefore be very useful to refine the general approach for future outbreaks of emerging diseases, but this remains work for the future.

In conclusion, a real-time GPFD surveillance system can be set up rapidly during an epidemic and is able to show the progress of the epidemic. Such an inexpensive system can be deployed even in resource-poor settings to track future influenza epidemics and pandemics and forecast their trajectories in near real-time.

## Materials and Methods

### Ethics approval

Ethics approval for the project was obtained from the institutional review board of the National University of Singapore.

### Recruitment, enrollment and inclusion criteria for GPFDs

We obtained e-mail addresses of GPFDs in Singapore from the College of Family Physicians Singapore (CFPS) and the directory of Pandemic Preparedness Clinics, a group of over 200 clinics registered with the Ministry of Health to manage influenza cases. In all, invitations were sent to 535 e-mail addresses. A series of road shows was also conducted at the CFPS to describe how ILI surveillance could help to track an epidemic. GPFDs who agreed to participate were also asked to extend the recruitment to their contacts.

Participating GPFDs had to be doctors registered with the Singapore Medical Council who worked at least three full days a week in a general practice or family medicine clinic in the community. Participation was purely voluntary and participating GPFDs were given the option to withdraw from the project at any time.

### Data submission and processing

Enrolled GPFDs were requested to submit returns on their work days by e-mail or facsimile by 2pm the following day. The data submitted comprised information on clinically diagnosed ARIs. Clinically, influenza is an acute respiratory infection. As a group, the ARIs may be defined as a clinical diagnosis of patients who present with new short-term (time from onset less than two weeks) respiratory symptoms of cough, rhinorrhœ a, nasal congestion and/or sore throat, which may or may not be accompanied by fever. The syndrome is usually though not exclusively associated with viral æ tiologies. The range of pathogens responsible for ARI besides influenza is described in a recent WHO paper [44]. A number of viruses cause a clinical illness which is difficult to distinguish from influenza, including respiratory syncytial virus, piconaviruses, parainfluenza, and adenovirus. These produce an influenza like illness [45]. The operational definition of ILI we then used in performing the analyses was an ARI exhibiting a fever of 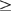
37.8°C; this approximates the definition used by the United States' Centers for Disease Control and Prevention, which defines ILI as an acute illness with cough and/or sore throat with a fever of 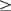
37.8°C, in the absence of a known cause other than influenza [46]. Other data elements collected in the data collection form (figure S1) included demographic, clinical, and antiviral treatment information.

### Mathematical modelling

Disease dynamics are modeled via a standard, stochastic compartmental model [47]–[50, *inter alios*], with daily increments and individuals passing through a series of unobserved classes corresponding to clinical stages of infection—Susceptible, Exposed (infected but not infectious), Infectious and Removed (recovered and subsequently immune, or deceased)—formulated by the equations
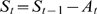


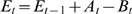


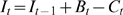


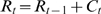
where 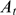, 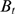, 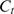 represent the number of people in the whole population newly infected, infectious, and removed, respectively. These are assumed to follow binomial distributions as follows
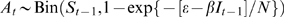


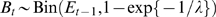


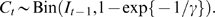
To be explicit, the infection model is formulated under the simplifying assumptions that:

1. infections are allowed to arise from importation at an assumptive constant rate 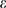 and from other local cases using the law of mass action, with 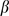 characterising the mixing and transmission probabilities of the local population, which as a first-order approximation is assumed to be homogeneous;
2. “importations” at rate 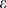 represent inhabitants of the country becoming infected via travel abroad or via travellers passing through Singapore, not of new immigrants entering the country infected;
3. the population size is taken to be fixed at 4.8M with no birth, death or genuine immigration or emigration during the epidemic (we ignored the fact that the official population size increased to 5M during the epidemic);
4. transition from exposed to infectious to removed is assumed to occur at constant rates 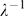 and 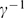, respectively;
5. per-capita rates (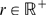) can be transformed to daily probabilities (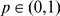) using the relationship 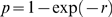; and
6. no parameters change with time.
  As with all models, the assumptions that go into ours can be criticised on biological, sociological and epidemiological grounds. Note that neither the parameters 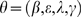 nor the states 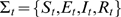 are known.
  The infection model is married to an observation model, namely that the (known) number of cases reported on day 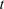 is 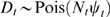 where 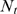 is the (known) number of GPFDs submitting reports on day 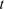 and
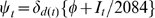
where 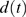 is the day of the week of day 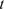 (Monday being 1 and so on). The parameters of the observation model are thus 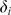 for 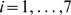
—the probability an infectious individual will seek medical attention on day of the week 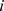
—and 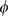, which is related to the “background” consulting rate for non-H1N1 ILIs. We took the differential reporting rates to be the same for H1N1 and non-H1N1 ILIs, so that 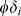 represents the typical number of ILIs per GPFD on day 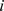 in the absence of the pandemic. There were 1480 GPFD in Singapore in 2001 [51], and the population grew 17% from 2001 to 2009, resulting in an estimated 1730 GPFDs in Singapore in 2009; 83% of patients attend these rather than polyclinics [51], and together these yield the divisor 2084; this permits the parameters to have a more natural interpretation (under the assumption that the participating GPFDs are representative) but is unneccessary for the analysis, as it functions as a mere rescaling of 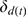. We artificially take the day of the week of public holidays to be a Sunday, with the normal week structure resuming the following day.
  The assumptions of the observation model are that:
7. consultations occur only when individuals are infectious;
8. consultations are conditionally independent;
9. per capita consulting probabilities for those infected with influenza A (H1N1-2009) are constant throughout the epidemic; and
10. overall consulting rates for other diseases that may be mistaken for influenza A (H1N1-2009) are also constant —excluding the day of week effect— i.e. there are no concurrent epidemics (an assumption subsequently supported by laboratory testing that suggested limited levels of co-circulating strains).

As before, the validity of these assumptions is open to debate.

In the original formulation, we forced 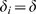 for all 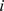, i.e. to be equal. In the middle of July, in response to the obvious variation over the week, we changed the constraint of the model to 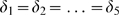 and 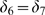. By mid-August, it was apparent that the day of the week effect needed to differ on each day of the week to attain a good fit. It is therefore the model without constraints that we present in this paper.

### Statistical methodology

The parameters of the model are estimated within the Bayesian statistical paradigm [52, for instance] in which semi-informative prior distributions are assigned to parameters and incoming data incorporated via the likelihood function to obtain a time series of posterior distributions for the parameters and unobserved state space.

Since the state space is unobserved, a statistical method called particle filtering [53], [54] is used to integrate over the possible realisations consistent with the daily observations. A series of 10 000 “particles” are created to which are associated parameter values and state space configurations generated from the prior distribution. Particles are iterated forward one day at a time via simulation of the state space, and the likelihood function calculated conditional on the trajectory of that particle and its associated parameter values. The likelihood function is then used to weight the particle. Particle degeneracy is overcome via resampling [54], while particle diversity is maintained via kernel smoothing [55]; the latter means that the resulting posterior distribution is approximate. The (approximate) posterior predictive distribution is derived by continuing the simulations beyond the last observation and weighting the resulting distribution via the particle weights at the last observation.

The particle filter algorithm proceeds as follows.

1. **Initialisation.** Set 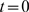. A set of 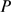 *particles* is drawn from the prior distribution for initial states 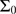 and parameters 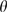. This prior distribution is described below, and is loosely based on preliminary findings from the literature. Particle 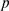 at time 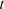 is a vector 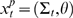 with associated weight 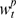. Initially, 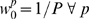.
2. **Iteration.** For each particle 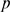, 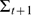 is drawn using Monte Carlo simulation from its conditional distribution given 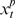.
3. **Weighting.** We then set 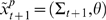. The likelihood contribution 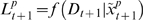 is then calculated. The weights are adjusted by setting 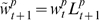 and then scaled so they sum to one:
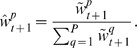

4. **Resampling.** Let 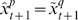 where 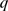 is drawn from the integers 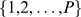 with probability proportional to 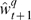, then setting 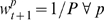.
5. **Kernel smoothing.** Let 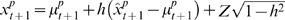 (with entries rounded to the nearest integer for state space values) where, following Trenkel *et al.* [55], we set 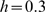, 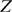 is generated from a multivariate Gaussian distribution with mean vector 0 and variance given by the variance-covariance matrix of 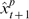 over all 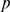, and 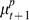 is the vector of means of 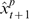 over all 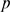 *if* the simulated value 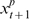 falls within the correct support *or* 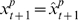 otherwise.
6. **Increment** 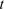 by one and repeat steps 2 onwards, until the current time is reached; thereafter to obtain the posterior predictive distribution repeat step 2 only (incrementing 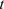) for as long as desired.

The algorithm provides the posterior distribution of any parameter, state or function thereof (such as the basic reproduction number, 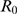, or the effective reproduction number, 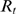, see e.g. [56], [57]) by taking a weighted average of this characteristic according to the posterior weights 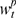 at the last observation time 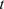. Here, only the posterior predictive distribution of the underlying states is of interest. Since the prior distributions taken were subjective (see below), the resulting posterior distributions are also subjective, and as a *caveat lector* we caution that our posterior distributions may differ from the reader's; for further information on subjective probability the reader is directed to the writings of de Finetti (e.g. [34]) or Lindley (e.g. [33]). For references on particle filtering and examples of its use in population dynamic modelling in ecology, see [53]–[55], [58]–[61].

The prior distributions used are given in figure 5. In setting these, we aimed to balance the need to supplement the information content of the sentinel data with relevant information from other sources, with the desire not to obliterate the signal from the data. We set the prior mean for the infection rate, 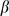, to be 1.2, with standard deviation 0.8. Combined with the prior distribution for the infectious period, this leads to a range for 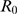 of 0 to around 6, i.e. more than spanning the range of estimates for historic pandemics. The prior distribution for the importation rate, 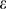, was derived from a crude extrapolation of the timeline of the first five weeks of importations to the country [62]. The prior distributions for the latent period and infectious period were modelled loosely on symptom onset after infection on an aeroplane [63] and a review of volunteer challenge studies [64]. The prior distributions for the background rate of non-pandemic ILIs (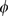) were based upon the clinical insight of the authors, and for the reporting probabilities from guesstimation, noting that it is common for employers or schools in Singapore to require a formal medical certificate before allowing staff or students off work or out of class. We conservatively forced 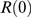 to be 0 since we did not know how the findings of studies in temperate countries [38] relating to prior exposure would extrapolate to the tropics; in this way, forecasts may be seen as worst case scenarios. The prior distributions for 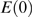 and 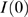 were derived from extrapolating the number of confirmed locally acquired cases.

Predictive error was assessed by taking the posterior distribution of absolute difference between forecasts and observations, averaged over a one-week time horizon, and then averaged to get the posterior mean prediction error.

All statistical routines were written by the authors using the R statistical programming language [65].

### Automation script

Modelling results were updated daily around 3pm to a website that could be publicly accessed [66]. This was automated using a bourne shell script that handled time, file transfer, archiving of previous forecasts, statistical processing, and positing of new output on the web. This was run on a unix web server using ISC's cron.

## Supporting Information

Figure S1 Data collection form.
(0.01 MB PDF)

Video S1 Animation of forecast average ILI per GPFD per day. Note the change in scale on the y-axis.
(0.81 MB SWF)

Video S2 Animation of forecast total nationwide ILI cases seeking medical attention. The day of week effect has been removed for clarity by treating all days as being Mondays. Note the change in scale on the y-axis.
(0.48 MB SWF)

Video S3 Animation of forecast proportion of population infected or recovered, including those not seeking medical attention. Note the change in scale on the y-axis.
(0.50 MB SWF)

## References

[1] WHO (2010). Influenza-like illness in the United States and Mexico. World Wide Web electronic publication.. http://www.who.int/csr/don/2009_04_24/en/index.html.

[2] WHO (2010). Pandemic (h1n1) 2009 - update 59. World Wide Web electronic publication.. http://www.who.int/csr/don/2009_07_27/en/index.html.

[3] Ministry of Health (Singapore) (2010). First confirmed case of influenza A (H1N1-2009) in Singapore. World Wide Web electronic publication.. http://www.moh.gov.sg/mohcorp/pressreleases.aspx?id=21914.

[4] Ministry of Health (Singapore) (2010). 26 confirmed new cases of influenza A (H1N1-2009). World Wide Web electronic publication.. http://www.moh.gov.sg/mohcorp/pressreleases.aspx?id=22202.

[5] Lee VJ, Chen MI, Chan SP, Wong CS, Cutter J (2007). Influenza pandemics in Singapore, a tropical, globally connected city.. Emerging Infectious Diseases.

[6] Lee VJ, Wong CS, Tambyah PA, Cutter J, Chen MI (2008). Twentieth century influenza pandemics in Singapore.. Annals of the Academy of Medicine, Singapore.

[7] Kanagaratnam K (1958). Influenza epidemic in Singapore, 1957.. Public Health.

[8] Huang QS, Lopez L, Adlam B (2007). Influenza surveillance in New Zealand in 2005.. Journal of the New Zealand Medical Association.

[9] Thompson WW, Comanor L, Shay DK (2006). Epidemiology of seasonal influenza: use of surveillance data and statistical models to estimate the burden of disease.. Journal of Infectious Diseases.

[10] Carrat F, Flahault A, Boussard E, Farran N, Dangoumau L (1998). Surveillance of influenza-like illness in France. the example of the 1995/1996 epidemic.. Journal of Epidemiology and Community Health.

[11] Fleming DM, Elliot AJ (2007). Lessons from 40 years' surveillance of influenza in England and Wales.. Epidemiology and Infection.

[12] Clothier HJ, Fielding JE, Kelly HA (2005). An evaluation of the Australian sentinel practice research network (ASPREN) surveillance for influenza-like illness.. Communicable Diseases Intelligence.

[13] Falcão IM, de Andrade HR, Santos AS, Paixão MT, Falcão JM (1998). Programme for the surveillance of influenza in Portugal: results of the period 1990–1996.. Journal of Epidemiology and Community Health.

[14] Gates P, Noakes K, Begum F, Pebody R, Salisbury D (2009). Collection of routine national seasonal influenza vaccine coverage data from GP practices in England using a web-based collection system.. Vaccine.

[15] Huang QS, Bandaranayake D, Lopez LD, Pirie R, Peacey M (2009). Surveillance for the 2009 pandemic influenza A (H1N1) virus and seasonal influenza viruses — New Zealand, 2009.. Morbidity and Mortality Weekly Report.

[16] Baker MG, Wilson N, Huang QS, Paine S, Lopez L (2009). Pandemic influenza A(H1N1)v in New Zealand: the experience from April to August 2009.. Eurosurveillance.

[17] Kelly H, Grant K (2009). Interim analysis of pandemic influenza (H1N1) 2009 in Australia: surveillance trends, age of infection and effectiveness of seasonal vaccination.. Eurosurveillance.

[18] Magnus D, Campbell J (2009). Pigs ears and the law of unintended consequences.. Archives of Disease in Childhood.

[19] Lipsitch M, Hayden FG, Cowling BJ, Leung GM (2009). How to maintain surveillance for novel influenza A H1N1 when there are too many cases to count.. Lancet.

[20] Chowell G, Nishiura H, Bettencourt LM (2007). Comparative estimation of the reproduction number for pandemic influenza from daily case notification data.. Journal of the Royal Society Interface.

[21] Cauchemez S, Valleron AJ, Boëlle PY, Flahault A, Ferguson NM (2008). Estimating the impact of school closure on influenza transmission from sentinel data.. Nature.

[22] Vynnycky E, Trindall A, Mangtani P (2007). Estimates of the reproduction numbers of Spanish influenza using morbidity data.. International Journal of Epidemiology.

[23] Vynnycky E, Edmunds WJ (2008). Analyses of the 1957 (Asian) influenza pandemic in the United Kingdom and the impact of school closures.. Epidemiology and Infection.

[24] Ferguson NM, Cummings DA, Cajka CFJC, Cooley PC, Burke DS (2006). Strategies for mitigating an influenza pandemic.. Nature.

[25] Nishiura H, Chowell G (2007). Household and community transmission of the Asian influenza A (H2N2) and influenza B viruses in 1957 and 1961.. Southeast Asian Journal of Tropical Medicine and Public Health.

[26] Vynnycky E, Pitman R, Siddiqui R, Gay N, Edmunds WJ (2008). Estimating the impact of childhood influenza vaccination programmes in England and Wales.. Vaccine.

[27] Hall IM, Hughes RGHE, Leach S (2007). Real-time epidemic forecasting for pandemic influenza.. Epidemiology and Infection.

[28] Lipsitch M, Riley S, Cauchemez S, Ghani AC, Ferguson NM (2009). Managing and reducing uncertainty in an emerging influenza pandemic.. New England Journal of Medicine.

[29] Doraisingham S, Goh KT, Ling AE, Yu M (1988). Influenza surveillance in Singapore: 1972–86.. Bulletin of the World Health Organization.

[30] Ministry of Health (Singapore) (2010). 95 new confirmed cases of influenza A (H1N1-2009). World Wide Web electronic publication.. http://www.moh.gov.sg/mohcorp/pressreleases.aspx?id=22340.

[31] Ministry of Health,Singapore (2009). Infectious diseases bulletin.. Epidemiol News Bull.

[32] Berger JO, Wolpert RL (1988). The Likelihood Principle.

[33] Lindley DV (2006). Understanding Uncertainty.

[34] de Finetti B (1974). The Theory of Probability.

[35] Gordon A, Ortega O, Kuan G, Reingold A, Saborio S (2009). Prevalence and seasonality of influenza-like illness in children, Nicaragua, 2005–2007.. Emerging Infectious Diseases.

[36] Viboud C, Alonso WJ, Simonsen L (2006). Influenza in tropical regions.. Public Library of Science Medicine.

[37] Chow A, Ma S, Ling AE, Chew SK (2006). Influenza-associated deaths in tropical Singapore.. Emerging Infectious Diseases.

[38] Katz J, Hancock K, Veguilla V, Zhong W, Lu XH (2009). Serum cross-reactive antibody response to a novel influenza A (H1N1) virus after vaccination with seasonal influenza vaccine.. Morbidity and Mortality Weekly Report.

[39] Richards FJ (1959). A flexible growth function for empirical use.. J Exp Bot.

[40] Hsieh YH, Cheng YS (2006). Real-time forecast of multiphase outbreak.. Emerg Infect Dis.

[41] Keyfitz N (1972). On future population.. J Am Stat Assoc.

[42] Taleb NN (2008). The Black Swan.

[43] Cowling BJ, Wong IO, Ho LM, Riley S, Leung GM (2006). Methods for monitoring influenza surveillance data.. Int J Epidemiol.

[44] WHO (2010). Acute respiratory infections. World Wide Web electronic publication.. http://www.who.int/vaccine_research/diseases/ari/en/print.html.

[45] Kelly H, Birch C (2004). The causes and diagnosis of influenza-like illness.. Australian Family Physician.

[46] CDC (2010). Influenza (flu) — flu activity. World Wide Web electronic publication.. http://www.cdc.gov/flu/weekly/fluactivity.htm.

[47] Bailey NTJ (1975). The Mathematical Theory of Infectious Diseases and its Applications.

[48] Anderson R, May R (1991). Infectious Diseases of Humans.

[49] Diekmann O, Heesterbeek JAP (2000). Mathematical Epidemiology of Infectious Diseases: Model Building, Analysis and Interpretation.

[50] Keeling M, Rohani P (2007). Modeling Infectious Diseases in Humans and Animals.

[51] Emmanuel SC, Phua HP, Cheong PY (2004). 2001 survey on primary medical care in Singapore.. Singapore Medical Journal.

[52] Lee PM (2009). Bayesian Statistics: An Introduction.

[53] Doucet A, Godsill S, Andrieu C (2000). On sequential Monte Carlo sampling methods for Bayesian filtering.. Statistics and Computing.

[54] Doucet A, De Freitas N, Gordon N (2001). Sequential Monte Carlo methods in practice.

[55] Trenkel VM, Elston DA, Buckland ST (2000). Fitting population dynamics models to count and cull data using sequential importance sampling.. Journal of the American Statistical Association.

[56] Roberts MG (2007). The pluses and minuses of *R*
_0_.. J Roy Soc Interface.

[57] Cauchemez S, Boëlle PY, Donnelly CA, Ferguson NM, Thomas G (2006). Real-time estimates in early detection of SARS.. Emerging Infectious Diseases.

[58] Cappé O, Godsill S, Moulines E (2007). An overview of existing methods and recent advances in sequential Monte Carlo.. Proceedings of the IEEE.

[59] Jègat C, Carrat F, Lajaunie C, Wackernagel H (2008). Early detection and assessment of epidemics by particle filtering.. geoENV VI – Geostatistics for Environmental Applications.

[60] Newman KB, Buckland ST, Lindley ST, Thomas L, Fernandez C (2006). Hidden process models for animal population dynamics.. Ecological Applications.

[61] Thomas L, Buckland ST, Newman KB, Harwood J (2005). A unified framework for modelling wildlife population dynamics.. Australian and New Zealand Journal of Statistics.

[62] Mukherjee P, Lim PL, Chow A, Barkham T, Seow E (2010). Epidemiology of travel-associated pandemic (H1N1) 2009 infection in 116 patients, Singapore.. Emerg Infect Dis.

[63] Moser MR, Bender TR, Margolis HS, Noble GR, Kendal AP (1979). An outbreak of influenza aboard a commercial airliner.. American Journal of Epidemiology.

[64] Carrat F, Vergu E, Ferguson NM, Lemaitre M, Cauchemez S (2008). Time lines of infection and disease in human influenza: a review of volunteer challenge studies.. American Journal of Epidemiology.

[65] R Development Core Team (2010). R: A Language and Environment for Statistical Computing. R Foundation for Statistical Computing, Vienna, Austria.. http://www.R-project.org.

[66] Cook AR, Lee HC (2010). Singapore influenza forecast. World Wide Web electronic publication.. http://www.stat.nus.edu.sg/staff/alexcook/flu/flu.html.

