# Supplementary material for: Real-Time Epidemic Monitoring and Forecasting of H1N1-2009 Using Influenza-Like Illness from General Practice and Family Doctor Clinics in Singapore
Source: PLoS One. 2010 Apr 14;5(4):e10036. doi: 10.1371/journal.pone.0010036 (PMC2854682; doi:10.1371/journal.pone.0010036)
Supplement: Figure S1 — Data collection form. (0.01 MB PDF) [file pone.0010036.s001.pdf]

Doctor's Name: **Clinic ID:** ____________

Clinic Stamp:

Date submitted: ____________

Data date: ____________

Total attendance seen: ____________

Fill in data for all acute respiratory infections (ARI) seen on above date.
Fax form to 62581527 or email this file to jimmy_ong@ttsh.com.sg.
Turn on CAPS LOCKS if entering data.

S/No	Sex (M / F)	Year of birth	Ethnicty (C / M / I / O)	S'porean or PR vs others (S / O)	Temperature (degrees C)	Oseltamivir or Zanamivir prescribed (Y / N)
